# Follicular Helper CD4^+^ T Cells in Human Neuroautoimmune Diseases and Their Animal Models

**DOI:** 10.1155/2015/638968

**Published:** 2015-08-02

**Authors:** Xueli Fan, Chenhong Lin, Jinming Han, Xinmei Jiang, Jie Zhu, Tao Jin

**Affiliations:** ^1^Department of Neurology, The First Hospital of Jilin University, Jilin University, Changchun 130021, China; ^2^Department of Neurobiology, Care Sciences and Society, Karolinska Institute, 14186 Stockholm, Sweden

## Abstract

Follicular helper CD4^+^ T (TFH) cells play a fundamental role in humoral immunity deriving from their ability to provide help for germinal center (GC) formation, B cell differentiation into plasma cells and memory cells, and antibody production in secondary lymphoid tissues. TFH cells can be identified by a combination of markers, including the chemokine receptor CXCR5, costimulatory molecules ICOS and PD-1, transcription repressor Bcl-6, and cytokine IL-21. It is difficult and impossible to get access to secondary lymphoid tissues in humans, so studies are usually performed with human peripheral blood samples as circulating counterparts of tissue TFH cells. A balance of TFH cell generation and function is critical for protective antibody response, whereas overactivation of TFH cells or overexpression of TFH-associated molecules may result in autoimmune diseases. Emerging data have shown that TFH cells and TFH-associated molecules may be involved in the pathogenesis of neuroautoimmune diseases including multiple sclerosis (MS), neuromyelitis optica (NMO)/neuromyelitis optica spectrum disorders (NMOSD), and myasthenia gravis (MG). This review summarizes the features of TFH cells, including their development, function, and roles as well as TFH-associated molecules in neuroautoimmune diseases and their animal models.

## 1. An Overview of Follicular Helper CD4^+^ T Cells

**Figure 1:**
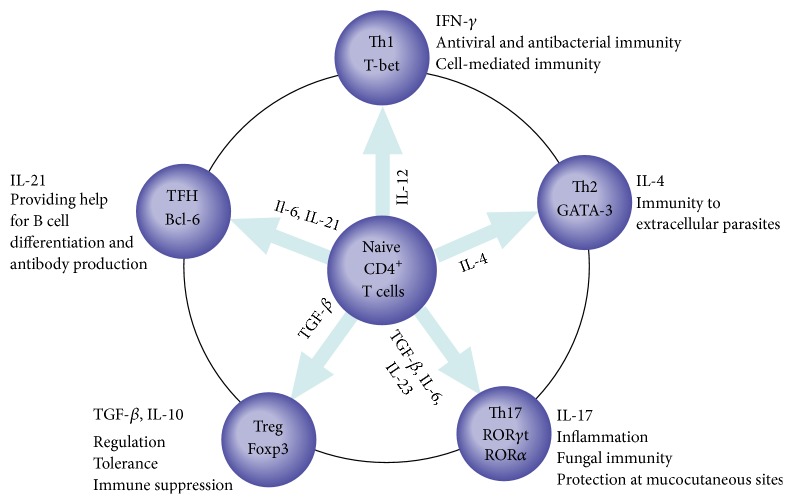
Effector subsets of CD4^+^ T cells: ontogenic and major cytokines, and roles in diseases. Naive CD4^+^ T cells differentiate into diverse effector subsets dependent on stimulatory cytokines in the microenvironment upon activation by pathogens. These stimulatory cytokines induce transcription factors expression of these subsets. IL-12 induces T-bet in the case of Th1 cells, IL-4 induces GATA3 in the case of Th2 cells, TGF-*β*, IL-6, and IL-23 induce ROR*γ*t and ROR*α* in the case of Th17 cells, TGF-*β* induces Foxp3 in the case of Treg cells, and IL-6 and IL-21 induce Bcl-6 in the case of TFH cells. Subsequently, different effector subsets produce distinct cytokines and acquire specialized effector function. Th1 cells produce IFN-*γ* associated with antiviral and antibacterial immunity and cell-mediated immunity, Th2 cells produce IL-4 associated with immunity to extracellular parasites, Th17 cells produce IL-17 associated with inflammation, fungal immunity, and protection at mucocutaneous sites, Treg cells produce TGF-*β* and IL-10 associated with regulation, tolerance, and immune suppression, and TFH cells produce IL-21 associated with providing help for B cell differentiation and antibody production. Bcl-6, B cell lymphoma 6; Foxp3, forkhead box p3; GATA-3, GATA-binding protein 3; IFN-*γ*, interferon-*γ*; IL-4, interleukin 4; IL-6, interleukin 6; IL-10, interleukin 10; IL-12, interleukin 12; IL-17, interleukin 17; IL-21, interleukin 21; IL-23, interleukin 23; ROR*γ*t, retinoid-related orphan receptor *γ*t; ROR*α*, retinoid-related orphan receptor *α*; T-bet, T-box transcription factor; TGF-*β*, transforming growth factor-*β*; TNF, tumour necrosis factor; Treg, T regulator.

CD4^+^ T helper (Th) cells play a critical role in adaptive immune response. After infection or vaccination, naive CD4^+^ T cells differentiate into diverse effector subsets of Th cells dependent on distinct cytokines and transcription factors [1–5] (Figure 1). These Th cell subsets possess respective effector function, for instance, the antiviral role of Th1 cells and the role in elimination of extracellular parasites of Th2 [2, 3] (Figure 1). Recently, follicular helper CD4^+^ T (TFH) cells, a specialized subset of CD4^+^ Th cells, have been identified as providing help for B cells in germinal center (GC) [6, 7]. GC is an important structure in B cell follicles of secondary lymphoid tissues, where B cells can differentiate into plasma cells and memory cells. TFH cells are distinguished from other Th cell subsets by anatomical location (germinal center), specialized expression of transcription factor B cell lymphoma 6 (Bcl-6), chemokine receptor CXC-chemokine receptor 5 (CXCR5), programmed death-1 (PD-1), CD40 ligand (CD40L), inducible costimulator (ICOS), SAP (signaling lymphocytic activation molecule associated protein), and secretion of interleukin 21 (IL-21) and interleukin 4 (IL-4) [8–10]. These TFH-associated molecules are vital for activation, differentiation, and survival of TFH cells and B cells [11]. In a word, TFH cells are pivotal to GC formation, providing help for affinity maturation, class switch recombination, and ultimate differentiation of B cells within GC [12]. The present review outlines the features of TFH cells and TFH-associated molecules in neuroautoimmune diseases, especially in multiple sclerosis (MS), neuromyelitis optica (NMO)/neuromyelitis optica spectrum disorders (NMOSD), and myasthenia gravis (MG) as well as their animal models, experimental autoimmune encephalomyelitis (EAE), and experimental autoimmune myasthenia gravis (EAMG).

### 1.1. Development of TFH Cells

**Figure 2:**
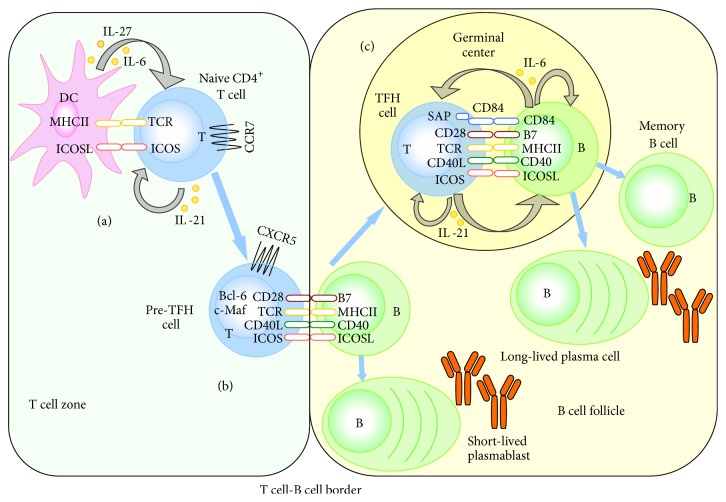
Multiple signals and steps for the generation of TFH cells. (a) Naive CD4^+^ T cells are activated when they encounter antigen presented cells-dendritic cells within T cell zone, and then these T cells move towards B cell follicles. (b) At the T cell-B cell border, activated T cells become pre-TFH cells, first interacting with cognate activated B cells, promoting either the differentiation of B cells into short-lived extrafollicular plasmablasts or the migration of B cells into follicles. (c) In germinal center, pre-TFH cells become GC TFH cells and provide help for B cell differentiation into plasma cells and memory B cells as well as antibody production. Cross-talk between TFH cells and cognate B cells involves a series of costimulatory molecules and cytokines, which are important for the function of TFH cells. Reciprocal signals provided by B cells are indispensable to sustain TFH cells. Bcl-6, B cell lymphoma 6; CCR7, CC-chemokine receptor 7; CD40L, CD40 ligand; CXCR5, CXC-chemokine receptor 5; DC, dendritic cell; ICOS, inducible costimulator; ICOS, ICOS ligand; IL-6, interleukin 6; IL-21, interleukin 21; IL-27, interleukin 27; MHC-II, major histocompatibility complex II; SAP, signaling lymphocytic activation molecule associated protein; TCR, T cell receptor.

It is generally accepted that the process of TFH cell differentiation is carried out in a multistage and multifactorial model [6, 11]. The first stage of TFH cell differentiation occurs in T cell zone of lymphoid tissues (Figure 2(a)). Naive CD4^+^ T cells are activated when they recognize dendritic cells (DCs) through peptide-MHC class II complexes and interact with DCs via the ligation of ICOS and ICOSL [13, 14]. Then these naive CD4^+^ T cells upregulate Bcl-6 and CXCR5, downregulate CC-chemokine receptor 7 (CCR7), and migrate towards B cell follicles [15, 16]. Meanwhile, IL-21 produced by these naive CD4^+^ T cells, accompanied with IL-6 and IL-27 produced by DCs, enhances Bcl-6 and c-Maf expression in naive CD4^+^ T cells [6]. Thus, the interplay between TCR signaling, ICOS, IL-21, IL-6, and IL-27 via control of CXCR5, Bcl-6, and other targets induces early stage of TFH cell differentiation. After that, these naive CD4^+^ T cells become pre-TFH cells (Bcl-6^+^CXCR5^+^ T cells). The second stage of TFH cell differentiation happens at the T cell-B cell border (Figure 2(b)). Here, pre-TFH cells first interact with cognate activated B cells, promoting either the differentiation of B cells into short-lived extrafollicular plasmablasts or the migration of B cells into follicles [13]. Although ICOS is a costimulatory molecule, it can also induce directional migration of pre-TFH cells after combining with ICOSL on activated B cells [6]. So ICOS-ICOSL binding is indispensable during this process. Furthermore, this process is a significant B cell-dependent course in which B cells offer antigen presentation and uninterrupted stimulation to promote full development of TFH cells [11]. The third stage of TFH cell differentiation involves the GC (Figure 2(c)). Within GC, pre-TFH cells finally differentiate into TFH cells that are also termed GC TFH cells. Pre-TFH cells and GC TFH cells, which are two phenotypically distinct stages in the development course of TFH cells, express analogical gene profiles. GC TFH cells express higher levels of Bcl-6, CXCR5, and ICOS than pre-TFH cells. GC TFH cells can prompt GC formation and provide help to B cells for affinity maturation, class switch recombination, and differentiation into memory B cells or plasma cells [17]. GC TFH cells interact with B cells dependent on stable T cell-B cell conjugates, which include CD40L/CD40, ICOS/ICOSL and CD28/B7, and CD4^+^ T cell-intrinsic signaling via SAP-associating receptors (CD84) [13]. In addition, B cells still serve as antigen presenting cells (APCs). Obviously, reciprocal signals provided by B cells play a significant role in sustaining TFH cells. In addition, IL-21 secreted by TFH cells prompts the final differentiation of TFH cells themselves, while IL-6 secreted by B cells is important for the maintenance of TFH cells. After the interaction between TFH cells and B cells in GC, the fate of TFH cells is unclear. Thus several questions are raised: (1) Are they apoptotic? (2) Do they become memory TFH cells? (3) Is there a cycle between pre-TFH cells and TFH cells [6]? Further investigations will be performed to verify the final fate of TFH cells.

### 1.2. The Function of TFH Cells and TFH-Associated Molecules

Pre-TFH cells help B cells to form short-lived extrafollicular plasma cells, which can produce low-affinity antibodies in the T cell-B cell border. GC TFH cells are crucial for humoral immune response against pathogens, because they are necessary in GC formation, affinity maturation, class switch recombination, and differentiation of B cells [18]. TFH cells express a lot of key molecules that are important for their function [19]. The roles of TFH-associated molecules are described as below.

#### 1.2.1. Bcl-6: B Cell Lymphoma 6

Bcl-6 is the master regulator transcription factor in TFH cells differentiation [6]. T cells with deficiency of Bcl-6 are unable to differentiate into TFH cells or sustain GC reactions, while Bcl-6 overexpression facilitates the expressions of TFH-associated molecules CXCR5 and PD-1 [20]. Besides, Bcl-6 represses numerous miRNAs and stabilizes the expression of CXCR5 [21]. Bcl-6 also represses Th1, Th2, and Th17 cell transcription factors T-box transcription factor (T-bet), GATA-binding protein 3 (GATA-3), and retinoid-related orphan receptor *γ*t (ROR*γ*t). However, the repression of Bcl-6 is not complete, as TFH cells sometimes express T-bet and GATA3 [22]. Another molecule, B lymphocyte induced maturation protein 1 (Blimp-1), directs the differentiation of CD8^+^ T cells, non-TFH CD4^+^ T cells, and plasma cells, which is a reciprocal antagonist of Bcl-6 and can inhibit TFH cell development [23]. So Bcl-6 can regulate all non-TFH cells differentiation through repressing Blimp-1 [24]. On the contrary, Blimp-1 also regulates the induction and function of Bcl-6 within TFH cells. The expression of Blimp-1 inhibits TFH cell formation, while Blimp-1 deficiency prompts the generation of TFH cells [23].

#### 1.2.2. CXCR5: CXC-Chemokine Receptor 5

CXCR5 is a highlighted surface marker of TFH cells and the most widely used marker to identify TFH cells [25]. Its specific ligand, CXC-chemokine ligand 13 (CXCL13), is mostly produced by B cells and follicle mesenchymal cells. Upregulation of CXCR5, combined with downregulation of CCR7, leads to the migration of TFH cells from the T cell zone to the B cell follicles (Figure 2), where they provide a help for B cell differentiation [13, 16]. The precise localization of TFH cells is important for proper generation and function of TFH cells. T cells with deficiency of CXCR5 result in fewer and smaller GC formation and decreased frequency of GC B cells [26].

#### 1.2.3. PD-1: Programmed Death-1

PD-1 is highly expressed by TFH cells, while its ligands (PD-L1 and PD-L2) are expressed by multiple cells including B cells [12]. It has been demonstrated that PD-1 is a negative regulator of the proliferation of CD4^+^ T cells. Thus, deficiency of PD-1 leads to an increase in TFH cells, but a decrease in IL-4 and IL-21 cytokine mRNA synthesis [27, 28]. Loss of PD-1 contributes to a reduction of long-lived plasma cells [27]. Overall, PD-1 signaling regulates the homeostasis and function of TFH cells and affects the formation of long-lived plasma cells.

#### 1.2.4. ICOS: Inducible Costimulator

ICOS, a member in the CD28 family of costimulatory molecules, is expressed on activated T cells, while its ligand ICOSL is expressed on B cells, macrophages, and other antigen presenting cells. A study demonstrated that ICOS deficiency impaired the development of TFH cells, so as to cause defect in GC formation and antibody production in response to primary and secondary immunization with sheep red blood cells in the spleen of mice [29]. Another study also showed that lack of ICOS resulted in poor GC formation and severe reduction of class-switched memory B cells [30]. Taken together, ICOS plays an essential role in TFH cells generation, GC formation, and antibody production.

#### 1.2.5. CD40L: CD40 Ligand

CD40L, the unique ligand of CD40, is present on the surface of TFH cells. Patients with mutations in CD40L have a reduced number of TFH cells. Furthermore, CD40L is a key factor for GC formation as well as B cell activation, proliferation, and survival in vitro and in vivo [6, 31]. The maintenance of TFH cells and GC B cells depends on CD40L-CD40 engagement [32]. Of particular interest, CD40L inhibits the differentiation of plasma cell [33]. To sum up, CD40L is critical to the generation of TFH cells and the fate of GC B cells.

#### 1.2.6. IL-21: Interleukin 21

IL-21, produced by activated CD4^+^ T cells and NKT cells, plays a major role in TFH cell survival and GC B cell proliferation, survival, and differentiation in GC [34]. IL-21 is the most potent cytokine for driving plasma cells differentiation in both mice and humans [35]. Additionally, IL-21 can induce both Blimp-1 and Bcl-6 expression on B cells in different conditions [36]. Interestingly, the effect of IL-21 in initiating GC B cell differentiation has substantial overlapping function with other cytokines such as IL-6 and IL-4 [37], indicating that these cytokines may have overlapping signal pathways. So the fate of B cells relies on the combination of IL-21 and additional signals from GC TFH cells [6]. Therefore, IL-21 has an elusive effect which needs to be further investigated.

#### 1.2.7. SAP: Signaling Lymphocytic Activation Molecule Associated Protein

SAP is an adaptor protein that binds to the cytoplasmic tails of signaling lymphocytic activation molecule (SLAM) family receptors. SAP is upregulated in TFH cells and is indispensable for GC development and T cell-B cell conjugates formation [6]. In the absence of SAP, pre-TFH cells migrate into GC less efficiently and lose their ability to stay in GC [38]. Consistently, another study showed that SAP-deficient mice were able to generate CXCR5^+^PD-1^+^ TFH cells, but these cells were unable to be retained in GC [17]. Taken together, it is demonstrated that SAP contributes to the terminal differentiation of pre-TFH cells into GC TFH cells.

There are other molecules produced or expressed by TFH cells such as SLAM family receptors and IL-4. SLAM family receptors, which consist of CD84, SLAM, SLAM family member 6 (SLAMF6), and SLAMF3, have the ability to bind SAP and form T cell-B cell conjugates, thus contributing to TFH cell differentiation and function [11]. IL-4 plays an important role in B cell survival and differentiation [6].

## 2. Human Circulating TFH Cells

Generally, TFH cells have been recognized by their anatomical location in secondary lymphoid tissues. However, it is difficult and impossible to get access to these lymphoid tissues in humans, so plenty of studies are performed with human peripheral blood samples [39]. It has been demonstrated that CD4^+^CXCR5^+^ T cells comprise a portion of circulating lymphocytes [37, 40]. These circulating CD4^+^CXCR5^+^ T cells express lower level of ICOS and PD-1 and hardly express Bcl-6 in comparison with TFH cells in lymphoid tissues [41]. When circulating CD4^+^CXCR5^+^ T cells are cultured in vitro, they are able to secret IL-21 and induce B cell differentiation. Thus, human circulating CD4^+^CXCR5^+^ T cells share a part of properties of TFH cells. But whether circulating CD4^+^CXCR5^+^ cells are counterparts of TFH cells in GC is uncertain. It has been reported that human circulating CD4^+^CXCR5^+^ T cells share functional capacities with TFH cells and seemingly stand for their circulating memory compartment [42]. Similar to TFH cells in GC, circulating CD4^+^CXCR5^+^ T cells induce the differentiation of naive and memory B cells to plasmablasts and promote class switching via IL-21 and ICOS [42]. Circulating CD4^+^CXCR5^+^ T cells also provide help to B cells through cognate interaction. Additionally, circulating CD4^+^CXCR5^+^ T cells express CCR7 and L-selectin, both of which are indicative of the property to migrate into secondary lymphoid tissues [42]. Circulating CD4^+^CXCR5^+^ T cells comprise two subsets: the CCR7^lo^PD-1^hi^ subset represents a TFH-cell precursor phenotype and CCR7^hi^PD-1^lo^ subset displays a resting state. The differentiation of these two subsets requires ICOS and Bcl-6 but not SAP, suggesting that circulating CD4^+^CXCR5^+^ T cells are predominantly generated before becoming fully mature effector TFH cells. Upon antigen reexposure, circulating CCR7^lo^PD-1^hi^ subset rapidly differentiated into mature TFH cells to promote GC formation and antibody response. So circulating CCR7^lo^PD-1^hi^ subset is representative of active TFH cells in secondary lymphoid organs and related to disease activity in autoimmune diseases. Consequently, blood CCR7^lo^PD-1^hi^CD4^+^CXCR5^+^ T cells stand for a new mechanism of immunological early memory [43]. Emerging evidence further demonstrates the hypothesis that there is a group of circulating memory TFH cells. Because circulating memory TFH cells not only share common molecular pathways as effector TFH cells during differentiation, but also effectively help B cells during antibody response, they are used as a marker to monitor TFH cells in autoimmune diseases [44].

More and more investigators have conducted experiments with respect to circulating memory TFH cells. These studies have defined circulating TFH cells by different markers. Circulating CD4^+^CXCR5^+^ T cells, CD4^+^CXCR5^+^ICOS^+^ T cells, CD4^+^CXCR5^+^ICOS^hi^ T cells, CD4^+^CXCR5^+^PD-1^+^ T cells, CD4^+^CXCR5^+^PD-1^hi^ T cells, and CD4^+^CXCR5^+^ICOS^+^PD-1^+^ T cells have been used to define TFH cells or the subsets of TFH cells in different diseases. Therefore, it is a serious problem in the immunology to clarify circulating TFH cells, whether these cells are able to represent circulating TFH cells. Furthermore, an authentic phenotype is required to define circulating TFH cells, which will be used in further investigations in immune related diseases.

Intriguingly, according to the expression of chemokine receptors, CXC-chemokine receptor (CXCR3) and CC-chemokine receptor (CCR6), circulating CD4^+^CXCR5^+^ T cells are classified into Th1-like (CXCR3^+^CCR6^−^), Th2-like (CXCR3^−^CCR6^−^), and Th17-like (CXCR3^−^CCR6^+^) subsets. It has been confirmed that Th2-like and Th17-like subsets potently induce naive B cells to produce antibodies via IL-21 whereas Th1-like TFH cells are unable to do so [42].

Although the precise role of circulating CD4^+^CXCR5^+^ T cells still remains a puzzle, the experiments on circulating CD4^+^CXCR5^+^ T cells may reflect partial perturbation of TFH cells in GC. A number of studies have demonstrated that circulating TFH cells may participate in the immune response of autoimmune diseases such as systemic lupus erythematosus [41, 45], rheumatoid arthritis [46], ankylosing spondylitis [47], bullous pemphigoid [48], and primary Sjögren's syndrome [49].

In conclusion, the exact phenotype, features, and roles of circulating CD4^+^CXCR5^+^ T cells are still unclear. Further investigations are required to clarify the conundrum.

## 3. TFH Cells in Neuroautoimmune Diseases

The dysregulation of TFH cells and TFH-associated molecules causes several human diseases [50]. The downregulation of TFH cells results in a series of immune deficiencies including X-linked lymphoproliferative disease and hyper-IgM syndrome, whereas the upregulation has been found in autoimmunity (i.e., systemic lupus erythematosus) and cancers [51, 52]. Neuroautoimmune diseases include multiple sclerosis (MS), neuromyelitis optica (NMO)/neuromyelitis optica spectrum disorders (NMOSD), and myasthenia gravis (MG). The exact pathogenesis of these diseases is not completely clear. Emerging data has suggested that TFH cells may be associated with development of these diseases (Table 1). The implications of TFH cells and TFH-associated molecules in neuroautoimmune diseases are summarized below.

### 3.1. TFH Cells and TFH-Associated Molecules in Multiple Sclerosis and Experimental Autoimmune Encephalomyelitis

MS is a progressive autoimmune disease caused by damage to the myelin and axons of brain and spinal cord in central nervous system (CNS) [53–55]. According to the multiple sclerosis phenotype descriptions in 1996, MS is classified into 4 clinical subtypes: relapsing remitting multiple sclerosis (RRMS), primary progressive multiple sclerosis (PPMS), secondary progressive multiple sclerosis (SPMS), and progressive relapsing multiple sclerosis (PRMS) [56]. To date, existing therapeutic drugs can only decrease disease relapse and improve clinical symptoms, revealing an urgent need for new therapies.

The accurate pathogenesis of MS is unknown. Previously, MS was considered as a T cell-mediated autoimmune disease [57, 58]; nonetheless, a lot of groups have shown that MS is an immune-mediated disorder involved with humoral and cellular immunity [55, 59–61], with infiltration of activated T cells and macrophages, dendritic cells, B cells, and plasma cells [55, 58, 59]. In addition, other significant hallmarks of MS cover synthesis of oligoclonal immunoglobulins and the presence of B cell clonal expansion in cerebrospinal fluid (CSF), which shows that B cells play an important role in MS [62]. Later, it has been demonstrated that meningeal B cell follicles in SPMS were related to severe pathological changes, rapid disease progression, and poor prognosis [63]. These two studies further declared that humoral immunity may participate in disease development.

TFH cells are essential to humoral response due to their roles in GC formation, B cell differentiation, and antibody production [6]. Recently, a study showed that there was an increased frequency of ICOS^+^ TFH cells in circulating CD4^+^CXCR5^+^ T cells of both RRMS and SPMS compared to healthy controls [64]. ICOS^+^ TFH cells, as an activated TFH cell subset, were correlated with disease progression in SPMS. What is more, the frequency of ICOS^+^ TFH cells was related to plasmablasts, suggesting that ICOS^+^ TFH cells may play a crucial role in B cell activation [64]. Another study found that SPMS patients had an increased gene expression of ICOS, IL-21, and IL-21R in purified CD4^+^ T cells [65]. In addition, the expression of ICOS was also increased in cells from the CSF of progressive MS patients [65]. Tzartos et al. showed that the expression of IL-21 was increased in CD4^+^ T cells of infiltrating acute and chronic active lesions compared to inactive lesions of CNS in MS patients [66]. Furthermore, the polymorphisms of IL-21R were associated with MS in a genetic study [67]. The level of IL-21 mRNA in peripheral blood of SPMS patients reduced after treatment with mitoxantrone [64]. Additionally, there was a decreased percentage of blood CXCR3^+^ Th1-like TFH cells in all subtypes of MS patients, while there was an increased percentage of blood CCR6^+^ Th17-like TFH cells in PPMS patients [64].

Experimental autoimmune encephalomyelitis (EAE) is a traditional animal model for MS, which can be induced by active immunization with myelin components or by adoptive transfer of myelin-reactive CD4^+^ T cells. It is accepted that EAE is a T cell-mediated immune disease with similar pathologic characteristics of MS. Hence, EAE is used to study the pathogenesis and therapy for MS [68]. A recent study found that there were lymphoid follicle-like structures within the meninges of progressive relapsing EAE as well as increased gene expression of CXCL13 in the CNS of EAE [69], indicating a pathogenic role of humoral immunity in EAE. CXCR5 mRNA was present in spinal cord mononuclear cells (MNCs) in some of the mice with EAE. Furthermore, most CNS infiltrating CXCR5^+^ cells were CD3^+^CD4^+^ T cells in spinal cord MNCs in EAE [70]. Nohra et al. pointed out that the polymorphisms of IL-21R were associated with EAE in a genetic study [67]. Another study showed that when IL-21 was administered before EAE induction, it reinforced inflammatory influx into the CNS and exacerbated the severity of EAE [67].

In summary, TFH cells may be involved in the formation of ectopic B cell follicles with GC in the meninges of SPMS patients and intrathecal immunoglobulins synthesis. In addition, CD4^+^ T cells that highly express CXCR5 and ICOS are assumed to be the most potent inducers of IgG production [71]. The dysregulation of TFH cell function and TFH-associated molecules, ICOS and IL-21, have likely taken part in the pathogenesis of MS. IL-21, the most important cytokine secreted by TFH cells, was increased both in peripheral CD4^+^ T cells and in CD4^+^ T cells of brain active lesions within MS patients. All the above suggests that TFH cells may play a vital role in the pathogenesis of MS. It is supposed that TFH cells can be considered as a marker for disease progression, severity, and prognosis. Consequently, these results also provide a novel therapeutic strategy for targeting TFH cells in the treatment of MS.

### 3.2. TFH Cells and TFH-Associated Molecules in Neuromyelitis Optica/Neuromyelitis Optica Spectrum Disorders

NMO is an autoimmune disease characterized by recurrent attacks of severe optic neuritis and transverse myelitis [72–74]. NMO spectrum disorders (NMOSD) are limited forms of NMO, including relapsing optic neuritis, recurrent transverse myelitis, and some special encephalopathic presentations [75]. Historically, NMO was considered to be a variant of multiple sclerosis. However, since anti-aquaporin 4 autoantibody (AQP4-Ab) was found mostly in NMO patients, while not in MS patients, our understanding of these two diseases has been markedly changed [76, 77]. Nowadays there is a lot of convincing evidence from human and animal experiments that AQP4-Ab plays a central role in the pathogenesis of NMO/NMOSD [78, 79]. It is hypothesized that AQP4-Ab binds to AQP4 which is mainly expressed on astrocytic end-feet in CNS and subsequently leads to damage to blood-brain barrier involving complement dependent astrocyte cytotoxicity, followed by recruitment of neutrophils and eosinophils, and cytokines secretion [80]. All of these finally result in oligodendrocyte death, myelin loss, and neuronal injury [81]. In general, NMO/NMOSD is a complicated neuroautoimmune disorder characterized with humoral immunity [78, 82]. To date, there have been a few reports about the roles of TFH cells and TFH-associated molecules in NMO/NMOSD. Lately, a study showed that the percentage of circulating CD4^+^CXCR5^+^PD-1^+^ T cells and the level of serum IL-21 were higher not only in NMOSD patients than in MS patients and healthy controls, but also in relapsing NMOSD patients than in remitting NMOSD patients, suggesting that TFH cells and IL-21 were related to disease activity [83]. Moreover, CD4^+^CXCR5^+^PD-1^+^ T cells population and serum IL-21 level were decreased after treatment with methylprednisolone in NMOSD patients [83]. However, the number of CD4^+^CXCR5^+^PD-1^+^ T cells was not related to AQP4-Ab titers in AQP4-Ab positive NMOSD patients. Consistent with this, another study showed that the release of IL-21 by peripheral blood mononuclear cell (PBMC) cultures was higher in NMO patients than in healthy controls and the level of IL-21 produced by peripheral blood CD4^+^ T cell cultures was positively correlated to expanded disability status scale (EDSS) score in NMO patients [84]. Besides, the levels of CXCR5 in both serum and CSF were increased in NMO patients compared with controls. B cell chemokine CXCL13, the ligand of CXCR5, plays an important role in the recruitment of CXCR5-expressing cells. It has been found that the CSF CXCL13 level was also correlated with disability of NMO patients [85]. Taken together, it is verified that there is a close association of TFH cells and TFH-associated molecules with disease activity of NMOSD. TFH cells also serve as a biomarker of NMO/NMOSD. But further study is needed to determine whether the TFH cells favor disease pathogenesis especially the generation of AQP4-Ab in NMO/NMOSD patients. It is also important to uncover whether TFH cells are a new therapeutic target in NMO/NMOSD patients.

### 3.3. TFH Cells and TFH-Associated Molecules in Myasthenia Gravis and Experimental Autoimmune Myasthenia Gravis

MG is a pathogenic autoantibody mediated neuroautoimmune disease characterized by a postsynaptic defect of neuromuscular transmission [86, 87]. Autoantibodies mainly contain antibodies against acetylcholine receptor (AChR) and those against muscle-specific tyrosine kinase (MuSK) [88] or lipoprotein receptor-related protein 4 (LRP4) and so on [89]. MG with anti-AChR antibody is the most common type [90]. Anti-AChR antibody combines with AChR blocking acetylcholine engagement with AChR, which subsequently induces complement-mediated damage to muscle fibers [91, 92]. The autoantibodies against MuSK and LRP4 impair AChR clustering, also resulting in the failure of neuromuscular transmission [93]. Although the exact trigger of autoimmunity in MG is still unclear, it is generally considered that the thymus is essential for the pathogenesis of MG [94]. Thymic lymphoid hyperplasia and thymoma are common pathological changes in MG patients. The hallmark of MG is the fatigability of skeletal muscle groups, including ocular, bulbar, and facial muscles and limbs [95]. The weakness is fluctuating, worsening with repeated activity and improving by rest [86]. Currently, the treatments for MG consist of anticholinesterase agents, immunotherapy composed of immune suppressions, plasma exchange and intravenous immunoglobulin, and thymectomy [96].

TFH cells efficiently induce antibodies generation by B cells, and there are more and more studies concerning the role of TFH cells in the pathogenesis of MG. It is reported that the frequency of CD4^+^CXCR5^+^ [97, 98], CD4^+^CD45RO^+^CXCR5^+^ [98], CD4^+^CXCR5^+^PD-1^hi^, and CD4^+^CXCR5^+^ICOS^hi^ T cells in the peripheral blood from MG patients was higher compared to healthy subjects. Significantly, there was a positive association between the percentage of CD4^+^CXCR5^+^ T cells and disease severity [97]. Also, the percentage of CD4^+^CXCR5^+^ICOS^hi^ or CD4^+^CXCR5^+^PD-1^hi^ T cells had a positive association with the levels of serum anti-AChR antibody [98]. Moreover, the frequency of CD4^+^CXCR5^+^ T cells was reduced after treatment in MG patients [97]. CXCL13 and its receptor CXCR5 are essential to form lymphoid follicles. It was found that the level of serum CXCL13 was increased in MG patients [99, 100] and positively correlated with disease severity and the frequency of circulating CD4^+^CXCR5^+^ICOS^hi^ T cells [98]. IL-21 is a vital cytokine for GC formation, TFH cell differentiation, and antibody production. It has been discovered that IL-21 mRNA expression in PBMCs was increased and positively related to the percentage of CD4^+^CXCR5^+^ICOS^hi^ T cells in MG patients [98].

Experimental autoimmune myasthenia gravis (EAMG) is a conventional model for MG. EAMG is induced by immunization with AChR from fish electric organs [101]. On account of the similar clinical and immunopathological traits with MG, EAMG is widely used to explore the mechanism and treatment of MG. It has been demonstrated that CD4^+^CXCR5^+^PD-1^+^ TFH cells and TFH-associated molecules, Bcl-6 and IL-21, in the spleen mononuclear cells of EAMG mice were upregulated. Furthermore, the level of serum anti-AChR antibodies was positively associated with the frequency of TFH cells in spleen [102]. Moreover, RNA interference targeting Bcl-6 in EAMG effectively ameliorated clinical severity with reduced frequency of TFH cells, decreased expression of Bcl-6 and IL-21, and low level of anti-AChR antibody [102]. Thus, TFH cells may participate in the pathogenesis and become a new therapeutic target for EAMG.

CD4^+^CXCR5^+^ T cells may be a marker to assess the disease activity and the therapeutical effect of medicines in MG. Meanwhile, TFH cells may be an alternative therapeutic target in MG.

## 4. Conclusion

TFH cells are considered to be involved in the pathogenesis of neuroautoimmune diseases. TFH cells and TFH-associated molecules might be the potentially useful targets for a novel therapeutic selection in neuroautoimmune diseases. Further studies are still needed to better understand the roles of TFH cells in these diseases, which will open a new avenue to explore the mechanisms of the autoimmune process in neuroautoimmune diseases.

## Figures and Tables

**Table 1:** TFH cells and TFH-associated molecules in neuroautoimmune diseases and their animal models.

Neuroautoimmune diseases	Changes of TFH cells and TFH-associated molecules	Relevance to the diseases	References
MS	↑ frequency of circulating CD4^+^CXCR5^+^ICOS^+^ T cells in RRMS and SPMS patients ↑ gene expression of ICOS, IL-21, and IL-21R in purified CD4^+^ T cells in SPMS patients ↑ ICOS expression in CSF cells in progressive MS patients ↑ IL-21 expression of CD4^+^ T cells in infiltrating active lesions of CNS ↓ frequency of blood CXCR3^+^ Th1-like TFH cells in MS patients ↑frequency of blood CCR6^+^ Th17-like TFH cells in PPMS patients	Positive correlation between circulating CD4^+^CXCR5^+^ICOS^+^ T cells and disease progression in SPMS	[64–66]

EAE	CXCR5 mRNA present in spinal cords CNS infiltrating CXCR5^+^ cells were CD3^+^CD4^+^ T cells in spinal cord MNCs		[70]

NMO/NMOSD	↑ frequency of circulating CD4^+^CXCR5^+^PD-1^+^ T cells ↑ serum level of IL-21 ↑ secretion of IL-21 in PBMC cultures ↑ serum and CSF levels of CXCR5	A correlation between circulating CD4^+^CXCR5^+^PD-1^+^ T cells and disease activity Positive correlation between the level of IL-21 produced by peripheral blood CD4^+^ T cells and EDSS score Positive correlation between CSF CXCL13 level and disease disability	[83–85]

MG	↑ frequency of circulating CD4^+^CXCR5^+^ T cells, CD4^+^CXCR5^+^CD45RO^+^ T cells, CD4^+^CXCR5^+^ PD-1^hi^ T cells, and CD4^+^CXCR5^+^ICOS^hi^ T cells ↑ serum level of CXCL13 ↑ expression of IL-21 mRNA in PBMCs	Positive correlation between circulating CD4^+^CXCR5^+^ T cells and disease severity Positive correlation between circulating CD4^+^CXCR5^+^ICOS^hi^ T cells and serum anti-AChR Ab concentration Positive correlation between circulating CD4^+^CXCR5^+^PD-1^hi^ T cells and serum anti-AChR Ab concentration Positive correlation between serum level of CXCL13 and disease severity Positive correlation between serum level of CXCL13 or expression of IL-21 mRNA in PBMCs and circulating CD4^+^CXCR5^+^ICOS^hi^ T cells	[97–100]

EAMG	↑ frequency of CD4^+^CXCR5^+^PD-1^+^ TFH cells and increased expression of Bcl-6 and IL-21 in spleens	Positive correlation between serum level of anti-AChR Abs and the frequency of TFH cells in spleen	[102]

AChR, acetylcholine receptor; Bcl-6, B cell lymphoma 6; CCR6, CC chemokine receptor 6; CNS, central nervous system; CSF, cerebrospinal fluid; CXCL13, CXC-chemokine ligand 13; CXCR3, CXC-chemokine receptor 3; CXCR5, CXC-chemokine receptor 5; EAE, experimental autoimmune encephalomyelitis; EAMG, experimental autoimmune myasthenia gravis; EDSS, expanded disability status scale; ICOS, inducible costimulator; IL-21, interleukin 21; IL-21R, IL-21 receptor; MG, myasthenia gravis; MNCs, mononuclear cells; MS, multiple sclerosis; NMO, neuromyelitis optica; NMOSD, neuromyelitis optica spectrum disorders; PBMC, peripheral blood mononuclear cell; PD-1, programmed death-1; RRMS, relapsing remitting multiple sclerosis; SPMS, secondary progressive multiple sclerosis.
